# Humanizing science: seven actions for PhD students to become next generation, future-proof scientists

**DOI:** 10.12688/openreseurope.15083.1

**Published:** 2022-10-13

**Authors:** Ingrid Valks, Dara Satrio, Angelica Reitsma, Katja Wolthers, Kris Dierickx, Kim Benschop, Dasja Pajkrt, OrganoVIR

**Affiliations:** 1 Amsterdam Medical Centre, Amsterdam, The Netherlands; 2 KU Leuven University, Leuven, Belgium; 3 Rijksinstituut voor Volksgezondheid en Milieu, Bilthoven, The Netherlands

**Keywords:** Personal Development, Early Stage Researchers, Next Generation Scientists, Innovative Training Network

## Abstract

PhD students, also referred to as the early stage researchers (ESRs), that are participating in the European Union’s Horizon 2020 consortium, OrganoVIR, have the ambition to become top scientists in virology with innovative, animal-free, research models; organoids. To achieve this ambition, developing more self-confidence and resilience was used to strengthen personal leadership needed in such professional role. Towards this purpose, seven actions have been selected that guide the ESRs through their PhD journey and help them elevate their career perspectives and employability in the international labor market. In this essay, we share the seven personal development actions that have been carried out by the ESRs in the OrganoVIR H2020 Innovative Training Network (ITN) project, with the goal of demonstrating how training human skills can contribute to innovation and collaboration in European research. This article is an effort by OrganoVIR’s Training and Education Committee to provide views on personal growth and leadership awareness.

## Plain language summary

This article highlights the importance of including a personal development program in a research training program, particularly, one that employs PhD students. A survey conducted by *Nature* (2019) shows that PhD students work for over 40 hours every week and that they ranked their work–life balance as a main concern throughout their PhD project. Additionally, academic pressure, uncertainty, and the amount of workload also play a role in mental health problems amongst PhD students. PhD students struggling with mental health problems are reluctant to seek treatment. This is caused by the fear of stigma or the fear of potential negative impact it might have on their future careers.

Supervised skill-building programs have shown to improve the lives of university students and to boost their professional influence ( Conley *et al.*, 2015). These programs have helped minimize mental health problems such as anxiety and depression and can improve social and emotional skills. Within the OrganoVIR Personal Development Plan, OrganoVIR’s PhD students, also known as early stage researchers (ESRs), are guided through seven actions to help them become the next generation of scientists. These actions include taking care of their personal well-being, achieving a better understanding of their purpose in life, discovering their own identity, having an excellent understanding of how values and beliefs impact internal and external communication, boost emotional competences, integrating insights in personal and professional life as well as experimenting with new leadership behavior, and recognizing and leading ethical dilemmas.

## Introduction

OrganoVIR is an EU Horizon 2020 Innovative Training Network (ITN) under the EU Horizon 2020 research program, coordinated by Dr. Katja Wolthers and Prof. Dasja Pajkrt. OrganoVIR’s training program trains PhD students or early stage researchers (ESRs) in the field of human organoids for virus research. When the OrganoVIR project was created, Wolthers and Pajkrt recognized that it is important for the next generation of scientists to develop their human skills (or personal skills) alongside their scientific skills. Therefore, at the start of the OrganoVIR project, three-skills sets were defined: 1) technical skills and academic skills to become an academic researcher in virus research with organoids through the scientific training program; 2) managerial skills through the newly developed and tailored pre-MBA; and, most importantly, 3) human skills through the BeyondU Personal Development Plan. Human skills, also called life skills or soft skills, are personal-oriented skills that require a mind–body–heart connection. These are skills that enable a person to think clearly, to collaborate harmoniously in teams and to lead people with compassion.

In this essay, we use the term “human skills” instead of “soft skills” as we believe that it provides a broad umbrella definition to describe a wide subset of characteristics to regulate emotions, act and react mindfully and be self-aware. *The Power of Time Off* is a consultancy firm for sustainable employability and conscious leadership. With the guidance of an experienced Personal Development coach from *the Power of Time Off*, the OrganoVIR Personal Development Plan (PDP) has been implemented to develop human skills of the ESRs. By doing so, they will increase their career perspectives and employability as well as ensure a balanced and healthy life.

### The new generation of scientists

A survey conducted by Nature (2019) reported that 76% of PhD students worked more than 40 hours per week and that 79% PhD students ranked their work–life balance as a main concern throughout their studies. Aside from their work–life balance, there are other factors that affect the mental health of PhD students. These include the pressure to publish, a strain in relationships with their advisors, uncertainty and financial insecurity, the competitive academic job market, and workload ( Levecque *et al.*, 2017; Mackie & Bates, 2019; Wyatt & Oswalt, 2013). The *Nature* survey also revealed that out of 6,320 PhD students only 36% of these students sought treatment for mental problems such as anxiety and depression. According to the Organisation for Economic Co-operation and Development (2015), the reluctance to seek help for mental problems amongst PhD students is caused by the fear of stigma or the negative impact on their future career.

Although technical skills are important to have in today’s labor market, having “human skills” such as empathy, adaptability, and critical thinking is what helps employees stand out. A study by Conley *et al.* (2015) have shown that skill-building interventions such as personal development programs can improve the lives of university students. In fact, Conley *et al*. (2015) proved that skill-building programs that include supervised practice are more successful in reducing anxiety, stress, depression, and in improving social and emotional skills. Taking all this into account, OrganoVIR provides the Personal Development Plan to all ESRs to help achieve a better performance, increase their professional influence by growing personal competences, and provide them with greater peace of mind.

During the intake, we used Google Forms to ask OrganoVIR’s 14 ESRs, who represent 13 nationalities, about their motivation to join OrganoVIR in an open question. As a result, we observed that there were three key factors that motivated these ESRs to join OrganoVIR: intellectual development and the opportunity to collaborate with an international team in academics and business, the opportunity to be mentored by experts from across Europe, and the training program that pays attention to self-development.

### Future-ready scientists go beyond science with the BeyondU PDP

The BeyondU PDP is a program that is fully committed to developing human skills, personal competences and practicing well-being as foundation skills for future-ready leaders in business and science. The program is based on the logical levels of Bateson and Dilts, an analysis and change model that provides insight into the different levels of communication, change and functioning and describes a systematic approach to change. Based on the hierarchy in the processes of learning, change, and communication formulated by Bateson (1972), Dilts (1996) defined six logical levels; purpose, identity, values and beliefs, emotional capabilities, behavior, and environment. The PhD students of OrganoVIR follow the modules in the PDP in the specific order recommended by Bateson and Dilts’ model.

Within the PDP, a seventh action is added: practicing well-being as a foundation skill to take leadership of our own mental well-being and vitality. Due to the typical 21st-century challenges such as a desire for finding meaning in life, rapid changes, overload of work and information, time pressure and the uncertainty of our role as humans with the rise of technology, we also need to take leadership of our own (mental) well-being and vitality ( Hougaard & Carter, 2018; Harari, 2019). The well-being modules align with the theory of both Scharmer (2009) and Judith (1987), who identify that humans need to connect on different intelligence levels such as physical, emotional, mental, and spiritual.

The PDP includes live, online and off-grid educational experiences or action learning. Action learning is learning from experience or harmonization of thinking and doing. Learning is an ongoing process that includes an open and curious mind, the ability to listen, ask questions and explore ideas. Action learning involves taking steps to learn through experience. Action learning has proven very effective in developing leadership and problem-solving methods and skills in practice. It has therefore become a regular part of the leadership development program of organizations.

Together with the coach of *the Power of Time Off*, we explored the seven actions in the BeyondU personal development plan which includes the following: 1) taking care of your personal well-being; 2) achieving a better understanding of your purpose in life; 3) discovering your own identity; 4) having an excellent understanding of how values and beliefs impact communication; 5) boost emotional competences; 6) integrating insights in personal and professional life as well as experimenting with new leadership behavior; and 7) recognizing and leading ethical dilemmas. These actions are described in more detail below.

### Action 1: practice well-being as a foundation skill

Practicing well-being as a foundation skill is key to improving personal resilience. Next-generation scientists must be physically, emotionally and mentally fit to overcome challenges, stress, anxiety and sometimes, loneliness in their way. Within the program, well-being modules are offered in the form of live masterclasses and virtual masterclasses (monthly e-vitality courses). The holistic approach of the well-being modules includes nature connection, meditation and breathing, nutrition, stress-relief and learning to relax. The ESRs state that the drive to participate in the wellbeing course is driven by the desire to release stress, to learn how to deal with anxiety, and to be more focused. After attending well-being courses, the ESRs self-reported an overall improvement in their mental wellbeing, happiness, concentration and focus, sleep pattern, and work relationships.

### Action 2: discover your purpose in life

Within the program, ESRs identified and explored their purpose and passion in life. Realizing their purpose can guide OrganoVIR’s ESRs through tough decisions and tough times and inspire them to move forward. By understanding their purpose, the ESRs will be able to identify situations that are not beneficial to them or that does not contribute to their purpose, and to avoid these situations or delegate tasks where possible. Furthermore, when ESRs realize their personal purpose is aligned with the purpose of OrganoVIR, it helps OrganoVIR’s ESRs not fall into burnout. This became apparent in times of uncertainties during the COVID-19 outbreak when many of the ESRs had limited access to their laboratories.

### Action 3: share your identity with the outside world

During the PDP, the ESRs learn to connect with their inner-voice and to master their physical voice. By speaking more concisely and clearly, the ESRs will be able to influence their audience more effectively; to convince them, to gain their attention during a presentation and to be recognized as an opinion leader. Personal coaching by an experienced personal development coach helps ESRs to turn weaknesses into strengths, showing them factors that hinder their growth and development. With this new skill, ESRs will be able to connect with themselves and have more impactful communication.

### Action 4: understand the impact of cross-cultural values and beliefs

It is important to understand, respond, and deal with how different people have different values on a macro and micro level, especially in an environment that harbors different cultures such as the OrganoVIR consortium. This fourth action minimizes communication gaps caused by cultural differences through increasing the ESR’s awareness of cultural values and the impact of behavior and intercultural communication in an international work environment.

OrganoVIR’s ESRs are also being guided to create awareness about their first interaction system, namely their family. More specifically, awareness is increased on behavior patterns that are occurred due to an individual’s upbringing. Having awareness on behavior patterns that are not accommodating to personal growth helps individuals to develop new, functional, and more productive patterns to communicate with others.

### Action 5: boost your emotional competences

Emotional competences, the ability to understand personal emotional reactions in work and life situations, is an essential factor of an individual’s personal and professional life. Having high emotional competences helps individuals the understanding of how emotions can impact productivity, work, and communication in specific scenarios. Individuals who have ambitions to have a professional influence and a healthy, happy work life will benefit from having emotional competences. 

Emotional competences are one of the defining characteristics of success in the workplace. To boost emotional competences, one must first begin with consciousness. Being aware of the present with a calm, focused, and clear mind will have a positive impact on the ESR’s physiology, psychology, and work performance. Second, one must have compassion for themselves and for others. Having compassion means the act or capacity for having sympathy for the suffering of yourself or others together with the wish to alleviate it. Third, one must have the ability to create meaningful connections including with their own selves. Creating meaningful connections will help to be good listeners and be more attentive, which will ultimately improve our self-esteem, happiness, and well-being.

### Action 6: work and lead from your future-self

To become a 21 ^st^-century leader in science, ESRs will need to embrace the challenges they face in today’s world. New, more human-focused, leadership starts with embodying well-being, developing personal competences, and leading from the future. During this module in the personal development plan, the ESRs reflect on their insights and learnings throughout their PDP journey. Sharing answers to questions such as “What have I already integrated in life and at work” and “What did I let go, what behavior am I experimenting with?”. Furthermore, the ESR’s behavior is the vehicle that takes them to what they would like to achieve in life. Since they are the driver of their behavior the ESRs are guided, during this PDP module, through a process in which they see themselves with a new perspective.

### Action 7: recognize moral dilemmas and demonstrate moral courage

OrganoVIR’s ESRs are encouraged to focus on their personal and professional environment. Within these environments, dilemmas will arise, and decisions must be made. To make a suitable decision, a good moral compass and moral courage is required. Therefore, ESRs are trained to identify and to lead conversations about ethical dilemmas in a seven-step-approach. To demonstrate moral courage, ESRs learn to bridge the gap between thinking and doing.

## Conclusion

Understanding people and creating connections, including with ourselves, is one of the most important aspects for PhD students to become a human and a future-proof scientist. PhD students who are well developed in their personal competences and who practice well-being as a foundation skill can take leadership of their personal and professional life. With its goal to develop the next generation of scientists to become the innovative leaders of tomorrow, OrganoVIR understands the need for a personal development program and has therefore incorporated the BeyondU Personal Development Plan into its training program. In a world of constant change, we must navigate our scientists to be ready for 21 ^st^ century challenges by providing a program that advances their human skills. Only then, when scientists have developed their human skills, will we be able to humanize science.

## Ethics and consent

Ethical approval and consent were not required.

## Data Availability

No data are associated with this article.
