# Humanizing science: seven actions for PhD students to become next generation, future-proof scientists

**DOI:** 10.12688/openreseurope.15083.2

**Published:** 2023-10-23

**Authors:** Ingrid Valks, Dara Satrio, Angelica Reitsma, Katja Wolthers, Kris Dierickx, Kim Benschop, Dasja Pajkrt, OrganoVIR

**Affiliations:** 1 Amsterdam Medical Centre, Amsterdam, The Netherlands; 2 KU Leuven University, Leuven, Belgium; 3 Rijksinstituut voor Volksgezondheid en Milieu, Bilthoven, The Netherlands

**Keywords:** Personal Development, Early Stage Researchers, Next Generation Scientists, Innovative Training Network

## Abstract

PhD students, also referred to as the early stage researchers (ESRs), that were participating in the European Union’s Horizon 2020 consortium, OrganoVIR, have the ambition to become top scientists in virology with innovative, animal-free, research models; organoids. To achieve this ambition, developing more self-confidence and resilience was used to strengthen personal leadership needed in such professional role. Towards this purpose, seven actions have been selected that guide the ESRs through their PhD journey and help them elevate their career perspectives and employability in the international labor market. In this essay, we share the seven personal development actions that have been carried out by the ESRs in the OrganoVIR H2020 Innovative Training Network (ITN) project, with the goal of demonstrating how training human skills can contribute to innovation and collaboration in European research. This article is an effort by OrganoVIR’s Training and Education Committee to provide views on personal growth and leadership awareness.

## Plain language summary

This article highlights the importance of including a personal development program in a research training program, particularly, one that employs PhD students. A survey conducted by *Nature* (2019) shows that PhD students work for over 40 hours every week and that they ranked their work–life balance as a main concern throughout their PhD project. Additionally, academic pressure, uncertainty, and the amount of workload also play a role in mental health problems amongst PhD students. PhD students struggling with mental health problems are reluctant to seek treatment. This is caused by the fear of stigma or the fear of potential negative impact it might have on their future careers.

Supervised skill-building programs have shown to improve the lives of university students and to boost their professional influence ( Conley *et al.*, 2015). These programs have helped minimize mental health problems such as anxiety and depression and can improve social and emotional skills. Within the OrganoVIR Personal Development Plan, OrganoVIR’s PhD students, also known as early stage researchers (ESRs), were guided through seven actions to help them become the next generation of scientists. These actions include taking care of their personal well-being, achieving a better understanding of their purpose in life, discovering their own identity, having an excellent understanding of how values and beliefs impact internal and external communication, boost emotional competences, integrating insights in personal and professional life as well as experimenting with new leadership behavior, and recognizing and leading ethical dilemmas.

## Introduction

OrganoVIR is an EU Horizon 2020 Innovative Training Network (ITN) under the EU Horizon 2020 research program, coordinated by Dr. Katja Wolthers and Prof. Dasja Pajkrt. OrganoVIR’s training program trains PhD students or Early Stage Researchers (ESRs) in the field of human organoids for virus research.

Over the years, human organoids have increasingly become an essential tool for virus research due to their ability to accurately mimic how human organs react to human viruses as well as antivirals. Understanding these interactions is a critical step to prevent viruses from spreading and ultimately, to develop treatments. Realizing its potential to transform the virology landscape, the OrganoVIR consortium trained ESRs to lead innovation in the field of organoids for virus research.

In the past, a high education qualification may have been sufficient for a PhD graduate’s employability ( de Weert, 2007), however, with changes such as increased globalization and increased job insecurity ( Forrier & Sels, 2003; Fugate *et al.*, 2004; Sung *et al.*, 2008), PhD graduates can no longer rely solely on their education qualification. Furthermore, in an age of advanced technology and automation, the ‘human’ jobs that will remain will require individuals with critical thinking and collaboration skills – skills that machines currently do not fully have ( Stirrett, 2017). PhD students will need to adapt to change and work towards achieving a competitive advantage over other graduates with similar academic backgrounds ( Tomlinson, 2012).

## The next generation of scientists

When the OrganoVIR project was created, Wolthers and Pajkrt recognized that it is important for the next generation of scientists to participate in a training program that covers the whole value chain, from laboratory to market. Developing and acquiring a combination of hard skills and soft skills will help PhD students to achieve a competitive advantage in the labour market ( Clarke, 2017; Crossman & Clarke, 2010). With training that focuses on developing their soft skills, the next generation of scientists will become confident and resilient individuals who lead with compassion and are able to operate within commercial settings.

Thus, at the start of the OrganoVIR project, three skill-sets were defined: 1) technical skills and academic skills to become an academic researcher in virus research with organoids through the scientific training program; 2) managerial skills through the newly developed and tailored pre-MBA; and, most importantly, 3) human skills through the BeyondU Personal Development Plan. Human skills, also called life skills or soft skills, are personal-oriented skills that require a mind–body–heart connection. These are skills that enable a person to think clearly, to collaborate harmoniously in teams, and to lead people with compassion ( Vasanthakumari, 2019).

In this essay, we will use the term “human skills” instead of “soft skills” as we believe that it provides a broad umbrella definition to describe a wide subset of characteristics to regulate emotions, act and react mindfully and be self-aware. These human skills, which can be developed outside formal learning and throughout life, are uniquely ‘human’ and are able to bridge the gap caused by robots and algorithms ( European Skills Agenda for Sustainable Competitiveness, Social Fairness and Resilience, European Commission, p.13, 2020). In this essay, we will be focusing on the third and final skill set; human skills developed through the Personal Development Plan (PDP).

## Going Beyond Science

During the intake, we used Google Forms to ask OrganoVIR’s 14 ESRs, who represent 13 nationalities, about their motivation to join OrganoVIR and what they would like to focus on in the next three years during their Personal Development Plan in two open questions.

**Figure 1.**
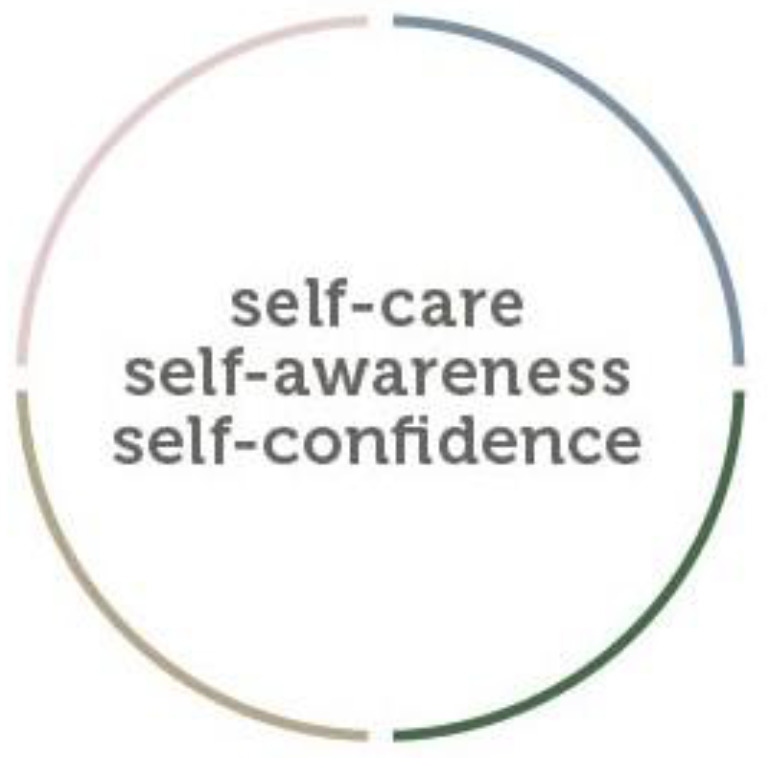
Three biggest personal changes ESRs want to make during their PhD journey.

As a result, we observed that there were three key factors that motivated these ESRs to join OrganoVIR: intellectual development and the opportunity to collaborate with an international team in academics and business, the opportunity to be mentored by experts from across Europe, and the training program that pays attention to self-development. Results showed that the ESRs would like to develop their self-confidence, understand themselves better, and maintain their well-being ( Figure 1).

Through collaboration with *The Power of Time Off*, a consultancy firm for sustainable employability and conscious leadership, OrganoVIR implemented the BeyondU Personal Development Plan (BeyondU PDP). The PDP is focused on helping the ESRs achieve better performance, increase their professional influence by developing personal competences, and provide them with greater peace of mind. Under the guidance of Valks, an experienced Personal Development coach and founder of *The Power of Time Off*, OrganoVIR trained ESRs to become confident and resilient leaders in the field of virology as a result, providing them with a competitive advantage in the international labor market.

The PDP is a blended program that integrates different approaches to providing support for the ESRs’ personal development which includes live, online through the e-learning platform and workshops, and off-grid educational experiences in nature. Throughout their PhD studies, OrganoVIR’s ESRs were also provided access to support groups, peer groups (for instance, a philosophical café initiated by one of the ESRs), and individual sessions with their personal development coach. During these individual sessions, the ESRs were able to address their personal challenges. These sessions have played an important role in supporting the ESRs throughout their project.

## The Personal Development Plan (PDP)

The BeyondU PDP is a program that is fully committed to developing human skills, personal competences, and practicing well-being as foundation skills for future-ready leaders in business and science. The program is based on the logical levels of Bateson (1972) and Dilts (1996), an analysis and change model that provides insight into the different levels of communication, change, and functioning and describes a systematic approach to change. Based on the hierarchy in the processes of learning, change, and communication formulated by Bateson (1972), Dilts (1996) defined six logical levels; purpose, identity, values and beliefs, emotional capabilities, behavior, and environment. The PhD students of OrganoVIR follow the modules in the PDP in the specific order recommended by Bateson and Dilts’ model.

Within the PDP, a seventh action is added: practicing well-being as a foundation skill to take leadership of our own mental well-being and vitality. Due to the typical 21st-century challenges such as a desire for finding meaning in life, rapid changes, overload of work and information, time pressure and the uncertainty of our role as humans with the rise of technology, we also need to take leadership of our own (mental) well-being and vitality ( Hougaard & Carter, 2018; Harari, 2019). The well-being modules align with the theory of both Scharmer (2009) and Judith (1987), who identify that humans need to connect on different intelligence levels such as physical, emotional, mental, and spiritual.

**Figure 2.**
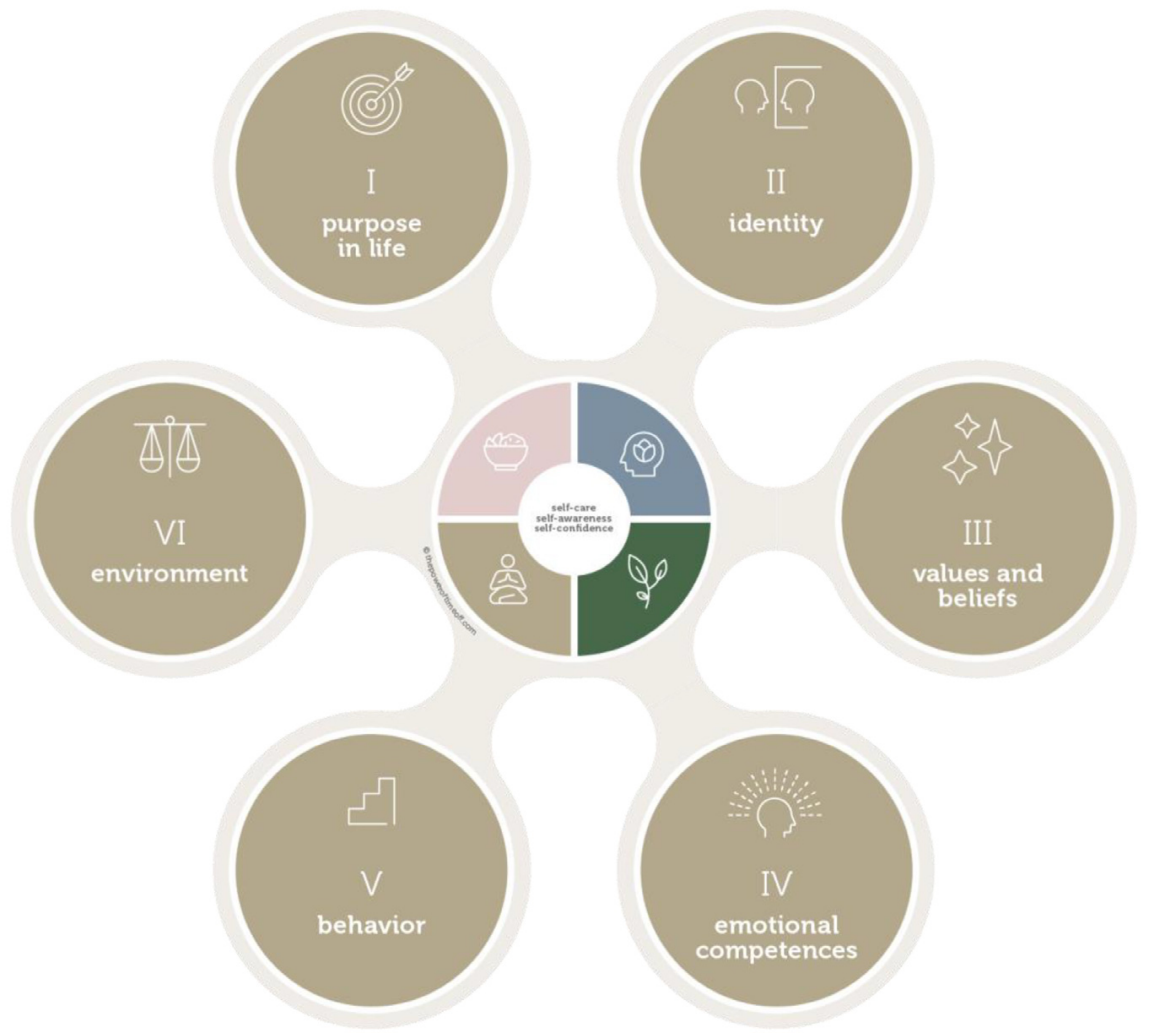
Humanity and Vitality Wheel by Valks in the BeyondU personal development program for OrganoVIR.

Together with the coach of *the Power of Time Off*, we explored the seven actions in the BeyondU personal development plan which includes the following: 1) taking care of your personal well-being; 2) achieving a better understanding of your purpose in life; 3) discovering your own identity; 4) having an excellent understanding of how values and beliefs impact communication; 5) boost emotional competences; 6) integrating insights in personal and professional life as well as experimenting with new leadership behavior; and 7) recognizing and leading ethical dilemmas. These actions are described in more detail below ( Figure 2).

### Action 1: Practice well-being as a foundation skill

It has been argued that personal well-being – defined by the Centers for Disease Control and Prevention (2018) as the presence of positive emotions, satisfaction with life, and positive functioning – is able to affect an individual’s ability to recover their mental health after facing challenges ( Stoffel & Cain, 2018). Practicing well-being as a foundation skill is key to improving personal resilience. The next generation of scientists must be physically, emotionally, and mentally fit to overcome challenges, stress, anxiety, and sometimes, loneliness in their way.

A survey conducted by Nature ( Lauchlan, 2019) reported that 76% of PhD students worked more than 40 hours per week and that 79% PhD students ranked their work–life balance as a main concern throughout their studies. Aside from their work–life balance, there are other factors that affect the mental health of PhD students. These include the pressure to publish, a strain on relationships with their advisors, uncertainty and financial insecurity, the competitive academic job market, and workload ( Levecque *et al.*, 2017; Mackie & Bates, 2019; Wyatt & Oswalt, 2013). The *Nature* survey also revealed that out of 6,320 PhD students, only 36% of these students sought treatment for mental problems such as anxiety and depression. According to the Organisation for Economic Co-operation and Development (2015), the reluctance to seek help for mental problems amongst PhD students is caused by the fear of stigma or the negative impact on their future careers.

A study by Conley *et al.* (2015) showed that skill-building interventions such as personal development programs can improve the lives of university students, reduce anxiety, stress, depression, and in improving social and emotional skills. Within the PDP, well-being modules were offered in the form of live masterclasses and virtual masterclasses (monthly e-vitality courses). The holistic approach of the well-being modules includes nature connection, meditation and breathing, nutrition, healthy lifestyle, stress relief, and learning to relax.

Following the completion of the OrganoVIR project and the PDP, ESRs were asked the fill in an evaluation survey. For one of the ESRs, the e-vitality courses reminded her of the importance of balancing her dream career in research and maintaining a healthy mindset. Additionally, to another ESR, the first action helped him not only to understand the importance of taking care of himself but also to address sources of issues from the workplace.

### Action 2: Discover your purpose in life

As human beings, each of us has the drive to find meaning and significance in our lives that enables us to endure challenges ( Frankl, 1963). Having a purpose motivates a person to dedicate their resources towards particular goals and to be more resilient to challenges and change ( Kashdan & McKnight, 2009). Under the guidance of experienced personal development coaches in a live masterclass, OrganoVIR’s ESRs identified and explored their purpose and passion in life.

According to Dhingra *et al.* (2021), when personal and organizational purposes are aligned, it will result in healthier, more resilient employees, stronger employee engagement, increased loyalty, and a higher willingness to recommend the company to others. Having a sense of purpose helps individuals to feel an increase in work gratification ( Bonebright *et al.*, 2000), life fulfillment, well-being and happiness ( Debats *et al.*, 1993; Zika & Chamberlain, 1992). This became apparent in times of uncertainties during the COVID-19 outbreak when many of the ESRs had limited access to their laboratories.

It is widely known that the COVID-19 pandemic has disrupted the scientific industry. As remote working grew popular for other occupations, studies showed a sharp decline in the amount of time scientists spent on research. For instance, in April 2020, a study found that scientists experienced a decrease of 7 hours in their laboratory compared to pre-pandemic ( Gao *et al.,* 2021). Additionally, it has been argued that the pandemic had impacted female scientists, ‘bench’ scientists (scientists who require workbenches for work in the laboratory), and scientists with young children the most compared to other groups of scientists ( Myers *et al.*, 2020).

Realizing their purpose can guide an individual through tough decisions and tough times and inspire them to move forward ( Brassey *et al.*, 2021). By understanding their purpose, the ESRs were able to identify situations that were not beneficial to them or that do not contribute to their purpose, and to avoid these situations or delegate tasks where possible.

### Action 3: share your identity with the outside world

The term *identity* refers to an individual’s traits, attitude, cognitive behaviors, and experiences ( Guenther *et al.*, 2020). There are two types of known identities; personal (otherwise known as individual) and social ( Vignoles, 2018). During the PDP, personal development coaches guide OrganoVIR’s ESRs to discover their identity through the masterclass ‘the power of your voice’, weekly online interactions, interactive assignments to help discover their identity, role model interviews, and finally, individual coaching calls.

By speaking more concisely and clearly, the ESRs were able to influence their audience more effectively; to convince them, to gain their attention during a presentation and to be recognized as an opinion leader. Personal coaching by an experienced personal development coach helps ESRs to turn weaknesses into strengths, showing them factors that hinder their growth and development. With this new skill, ESRs were able to connect with themselves and have more impactful communication.

Within the third action, OrganoVIR’s ESRs were taught the following lessons;

1. To become more aware of their proudest achievements, the things they like about themselves, and the things that give them joy. To listen to their inner voice and allow their senses to connect and build deeper human relationships, even through their computer screen during the pandemic.
2. To identify the most suitable tone of voice for themselves.
3. To become more self-confident and to step out of their comfort zone.

The aforementioned lessons were summarized based on answers provided by OrganoVIR’s ESRs. Answers were provided anonymously through an evaluation survey.

### Action 4: understand the impact of cross-cultural values and beliefs

Within an international environment, it is important to understand that different people have different values on a macro and micro level. Every country has their own special cultural thinking and values, otherwise known as a set of beliefs that serves as a guideline in an individual’s life that affects the way an individual evaluates others and certain events in their lives ( Kesberg & Keller, 2018; Zhao *et al.*, 2020). Hofstede’s work, in which he conceptualized how 40 countries and cultures differ ( Hofstede, 1983), is perhaps the best example to observe the differences of values among different cultures.

Often, miscommunications can occur during a cross-cultural interaction due to cultural gaps that neither parties are aware of ( Carté & Fox, 2008). The fourth action within the PDP minimized communication gaps caused by cultural differences through increasing the ESR’s awareness of cultural values and the impact of behavior and intercultural communication in an international work environment. During the masterclass ‘Social patterns, cultural beliefs and the impact on our communication’, which was carried out virtually due to the pandemic, ESRs were invited to take a closer look at values and beliefs in two levels; macro and micro.

Within the macro level, experienced PDP coaches guided the ESR to decode their own culture and their peer’s cultures. During the online masterclass, the ESRs were encouraged to answer the question ‘how does your, and others, culture communicate, persuade, trust, evaluate, disagree, decide, lead, and plan?’. Often, we are not fully aware of our cultural patterns and therefore we are not always aware of the impact of our behaviour on our foreign co-workers. The culture map of Erin Meyer (2014) was used throughout the masterclass as it focuses on how the world’s most successful leaders navigate the complexities of cultural differences in a multicultural environment.

Moreover, on a micro level, the ESRs delved into their first interaction system, namely their family. During this masterclass, the ESRs analyzed their background and how it formed their identity and to recognize patterns and behaviors that were helpful to their personal growth.

Upon completion of the fourth action, OrganoVIR’s ESRs anonymously reported the following lessons; 1) the ability to comprehend the differences between how high and low context culture communicates, 2) to be aware of the impact of their own communication style, and 3) motivation to become more assertive and confident in expressing their opinions.

### Action 5: boost your emotional competences

Emotional intelligence, also known as emotional competence, is the ability to understand personal and impersonal emotions, to discriminate between different emotions, and to use emotional information as a guide to think and behave ( Srivastava, 2013). Having high emotional intelligence can help individuals increase their productivity, ability to manage stress, and communication in specific scenarios ( Serrat, 2017; Sinha & Sinha, 2007). Individuals who have ambitions to have a professional influence and a healthy, happy work life will benefit from having emotional competences.

Emotional competences are one of the defining characteristics of success in the workplace. To boost emotional competences, one must first begin with consciousness. Being aware of the present with a calm, focused, and clear mind will have a positive impact on the ESR’s physiology, psychology, and work performance. Second, one must have compassion for themselves and for others. Having compassion means the act or capacity for having sympathy for the suffering of yourself or others together with the wish to alleviate it. Third, one must have the ability to create meaningful connections including with their own selves. Creating meaningful connections will help to be good listeners and be more attentive, which will ultimately improve our self-esteem, happiness, and well-being.

Much like the previous actions, the masterclass for the fifth action could not take place live due to the pandemic and therefore was carried out online. As a result, Valks provided the ESRs with weekly online learning content about Emotional Intelligence. Additionally, the ESRs were given the opportunity to participate in online retreats provided by the power of time off. The holistic approach of the virtual retreat calendar includes three courses to develop emotional intelligence, manage vitality and improve work-life balance: 1) mind training, 2) nature connection, and 3) stress relief and (learn to) relax.

### Action 6: work and lead from your future-self

To become a 21 ^st^ century leader in science, ESRs will need to embrace the challenges they face in today’s world. New, more human-focused, leadership starts with embodying well-being, developing personal competences, and leading from the future. During this module in the personal development plan, the ESRs reflect on their insights and learnings throughout their PDP journey. Sharing answers to questions such as “What have I already integrated in life and at work” and “What did I let go, what behavior am I experimenting with?”.

Furthermore, the ESR’s behavior is the vehicle that takes them to what they would like to achieve in life. Since they were the initiator of their behavior, the ESRs were guided, during this PDP module, through a process in which they see themselves with a new perspective.

### Action 7: recognize moral dilemmas and demonstrate moral courage

Through the final action in the PDP, OrganoVIR’s ESRs were encouraged to focus on their personal and professional environment. Within these environments, dilemmas will arise, and decisions must be made. To make a suitable decision, a good moral compass and moral courage is required. Therefore, ESRs were trained to identify and to lead conversations about ethical dilemmas in a seven-step-approach ( Loyens *et al.*, 2008). To demonstrate moral courage, ESRs learn to bridge the gap between thinking and doing.

The seventh action of the PDP is conducted in two sessions of masterclasses. In the first session, a step-by-step plan for moral inter-vision is provided to move from thinking and talking towards doing in difficult situations. The step-by-step plan or the moral inter-vision model is a tool that organizes and provides insight into the decision-making process in difficult situations and thus ensures that the conversation goes beyond exchanging experiences and giving spontaneous comments.

The model consists of 7 steps or 7 questions and forces some tempo delay, creating space to question the obvious. The model certainly also has room for emotions. An emotion is an 'advocate', an emotion indicates that something is at stake that we consider important and a response is needed.

The final session completes the circle of the BeyondU leadership program. Our purpose, identity, values, beliefs and emotional competences influence how we experience difficult situations. With the help of this masterclass, OrganoVIR’s ESRs discovered and learned how to deal with ethical dilemmas and to arrive at a moral solution.

Upon evaluation, 4 out of 14 ESRs claimed that the seventh action helped them recognize the importance of addressing sources of issues in the workplace and to demonstrate the courage to implement learnings from this action.

## Conclusion

Understanding people and creating connections, including with ourselves, is one of the most important aspects for PhD students to become a human and a future-proof scientist. PhD students who are well developed in their personal competences and who practice well-being as a foundation skill can take leadership of their personal and professional life. With its goal to develop the next generation of scientists to become the innovative leaders of tomorrow, OrganoVIR understands the need for a personal development program and has therefore incorporated the BeyondU Personal Development Plan into its training program. In a world of constant change, we must navigate our scientists to be ready for 21 ^st^ century challenges by providing a program that advances their human skills. Only then, when scientists have developed their human skills, will we be able to humanize science.

## Ethics and consent

Ethical approval and consent were not required.

## Data Availability

No data are associated with this article.
