# Supplementary material for: Humanizing science: seven actions for PhD students to become next generation, future-proof scientists
Source: Open Res Eur. 2023 Oct 23;2:119. Originally published 2022 Oct 13. [Version 2] doi: 10.12688/openreseurope.15083.2 (PMC10611947; doi:10.12688/openreseurope.15083.2)
Supplement: Peer review history

# Reviewer report: Reviewer response for version 2

**DOI:** 10.21956/openreseurope.17990.r35766

**Published:** 2023-11-06

**Authors:** Kim Brown

**Affiliations:** 1 University of Otago, Dunedin, Otago, New Zealand

I would like to approve this version and thank the authors for their attention and willingness to incorporate feedback.

Is the argument persuasive and supported by appropriate evidence?

Partly

Is the work clearly and cogently presented?

Partly

Is the topic of the essay discussed accurately in the context of the current literature?

Partly

Does the essay contribute to the cultural, historical, social understanding of the field?

Partly

Reviewer Expertise:

Doctoral education; transferable skills; inclusion.

I confirm that I have read this submission and believe that I have an appropriate level of expertise to confirm that it is of an acceptable scientific standard.

# Reviewer report: Reviewer response for version 2

**DOI:** 10.21956/openreseurope.17990.r35768

**Published:** 2023-10-27

**Authors:** Sara Cotterall

**Affiliations:** 1 School of Linguistics and Applied Language Studies, Victoria University of Wellington, Wellington, Wellington, New Zealand

I have read the revised article and am impressed at the extent to which the authors have improved the original draft.

 Although I couldn't read Figure 2 because it was too small, I appreciated that they justified their focus on the ESR's personal skills by including reference to aspects of their research context and research lives. This, for me, was essential to contextualise the study. I also appreciate that they included one or two examples of participants' reflections on the programme and how it had helped them.

 The only suggestion I would make is that they acknowledge how out of date Hofstede's work on cultural difference in the workplace is, and how discredited it has become. They could do this, for example, by saying something like - "although it has been criticised by some, Hofstede's original finding that...holds true" - or something like that.

Is the argument persuasive and supported by appropriate evidence?

Partly

Is the work clearly and cogently presented?

Yes

Is the topic of the essay discussed accurately in the context of the current literature?

Partly

Does the essay contribute to the cultural, historical, social understanding of the field?

Partly

Reviewer Expertise:

Doctoral education, Higher education, Language teacher education, Language learner autonomy, Language learning strategies

I confirm that I have read this submission and believe that I have an appropriate level of expertise to confirm that it is of an acceptable scientific standard.

# Reviewer report: Reviewer response for version 1

**DOI:** 10.21956/openreseurope.16311.r32326

**Published:** 2023-06-19

**Authors:** Kim Brown, Rachel Spronken-Smith

**Affiliations:** 1 Higher Education Development Centre, University of Otago, Dunedin, Otago, New Zealand; 2 University of Otago, Dunedin, Otago, New Zealand

1. This article could do more to clearly establish why, within the field of doctoral science study, it is beneficial to include a personal development program. The program sounds very interesting, but as noted by previous reviewers, more literature is available to robustly ground the assertions made in the article. Please cite Lauchlan (2019) as the author of the Nature survey report.
2. I agree that the term ‘human skills’ feels somewhat disconnected to the aims suggested. While the company that delivers the program uses the term ‘human skills’, within the field of doctoral research, ‘transferable skills’ is more commonly used. In this case, the particular subset of transferable skills being discussed are personal competencies and wellbeing skills. The research of Senekal, Munnik and Frantz (2022) ^1^ provides some guidance on personal resourcefulness as part of expected doctoral attributes, and is worth considering.
3. It is not clear in the article whether authors are justifying their decision to include a personal development programme (which I applaud on a personal level), or whether they are arguing that such a program should be part of doctoral education for scientists in the 21 ^st^ century, for which they do not present sufficient evidence. The reporting of the program switches verb tense between Action 2 and Action 3, suggesting that perhaps the program was underway but not complete at the time of writing? Related to this, it is unclear how actions 2, 4 and 5 are actually developed – what are the activities? Some clarity on the argument would help readers decide on whether sufficient evidence has been provided for the claims made in the conclusion.
4. The article certainly has potential to contribute to the cultural and social understanding of the field of personal development and wellbeing during doctoral study within the particular historical context that we find ourselves, pre- and post-pandemic. Inclusion of a greater range of data (participants’ quotes as suggested) would enhance the contribution this essay can make. If the authors have the capacity to do so, a longitudinal study of the cohort would be particularly helpful to the field of doctoral academic development.

Is the argument persuasive and supported by appropriate evidence?

Partly

Is the work clearly and cogently presented?

Partly

Is the topic of the essay discussed accurately in the context of the current literature?

Partly

Does the essay contribute to the cultural, historical, social understanding of the field?

Partly

Reviewer Expertise:

Doctoral education; transferable skills; inclusion.

We confirm that we have read this submission and believe that we have an appropriate level of expertise to confirm that it is of an acceptable scientific standard, however we have significant reservations, as outlined above.

## References

[1] : A systematic review of doctoral graduate attributes: Domains and definitions. *Frontiers in Education* .2022;7: 10.3389/feduc.2022.1009106 10.3389/feduc.2022.1009106

# Reviewer report: Reviewer response for version 1

**DOI:** 10.21956/openreseurope.16311.r32325

**Published:** 2023-06-13

**Authors:** Basil Cahusac de Caux

**Affiliations:** 1 American University of the Middle East, Egaila, Kuwait

This essay is a welcome attempt at raising awareness of the needs of doctoral candidates beyond the hard skills they develop through (multi)disciplinary research and supervision. However, it would benefit from a number of amendments:

 1. The work should be more concretely grounded in the literature on doctoral candidates. There are numerous papers, monographs, and edited books that deal specifically with the kinds of skills that doctoral candidates should develop, ways to develop such skills, and the necessity of fostering these skills in the first instance.

 2. Reconsider your use of the term 'human skills', as it is too broad and has no real precedent in the literature. I recommend substituting it with the term 'transferable skills', as it represents practically the same set of skills you describe in your essay.

 3. Given the small sample size (14 ESRs), it would be nice if you dedicated some space to the voices of your cohort. This would not only provide the reader with qualitative insights, but would also be good use of data that you have probably already generated. Doing this would lend more weight to the claims you make regarding 'self-reported [...] improvement in their mental wellbeing, happiness, concentration', etc.

 4. While you hint at the impact of the pandemic, due to the wholesale closure of labs and research facilities, it would be remiss of you not to mention the impact of the pandemic on 'the new generation of scientists'. There is plenty of literature on this. You can consult journals such as The International Journal of Doctoral Studies and edited books published by Springer (e.g. Research and Teaching in a Pandemic World).

 In addition to the above, there are a number of minor points that can be addressed. For instance, mention the year of publication immediately after the Bateson and Dilts citations (page 3). Add a visual representation of the Bateson (1972) and Dilts (1996) models to page 4. I also recommend adding a reference/ citation upon first mention of Action learning. Action 5 reads like a textbook description of mindfulness...is it? If so, cite one or two sources in the literature on mindfulness and doctoral candidates. Please also expand on Action 7, as it is too short in its current form. Please also use terminology consistently throughout your essay. For instance, you refer to your cohort as ESRs (early stage researchers) in most sections of the essay, but in the Conclusion you switch to PhD students. I would unify these two terms (as ESRs or PhD students -- the choice is yours).

 Lastly, there are some grammatical and stylistic issues that can be easily addressed by proofreading the essay.

Is the argument persuasive and supported by appropriate evidence?

Partly

Is the work clearly and cogently presented?

Partly

Is the topic of the essay discussed accurately in the context of the current literature?

Partly

Does the essay contribute to the cultural, historical, social understanding of the field?

Partly

Reviewer Expertise:

Sociology of Higher Education, Sociology of Language, Doctoral Studies

I confirm that I have read this submission and believe that I have an appropriate level of expertise to confirm that it is of an acceptable scientific standard, however I have significant reservations, as outlined above.

# Reviewer report: Reviewer response for version 1

**DOI:** 10.21956/openreseurope.16311.r31480

**Published:** 2023-06-01

**Authors:** Liezel Frick

**Affiliations:** 1 Department of Curriculum Studies, Stellenbosch University, Stellenbosch, Western Cape, South Africa

The article presented here is timely and relevant to the researcher careers and development community at large. The comments provided are aimed at helping the authors develop this work towards a more defendable contribution.

1. There is a growing body of literature that could support the argument which seems to have not been consulted - see for example the work of LERU working reports and the Wellcome Trust within grey literature, and the work of Kirsi Pyhalto and colleagues on researcher careers in STEM areas, as well as that of Karri Holley. 
2. The notion of 'action learning' is currently under-theorised, while there is a wealth of literature emanating from both the managements sciences and education on the topic. 
3. In order to make sense of the findings, readers would benefit from more detail on how the data was construed and interpreted in order to get to the seven actions presented here. This would make the study more defendable and rigorous. 
4. How does the BeyondU PDP programme compare/differ to other similar programmes? What are the shortcomings of the programme? What are possible implications for policy, practice and future research?

Is the argument persuasive and supported by appropriate evidence?

Partly

Is the work clearly and cogently presented?

Partly

Is the topic of the essay discussed accurately in the context of the current literature?

Partly

Does the essay contribute to the cultural, historical, social understanding of the field?

Partly

Reviewer Expertise:

Doctoral education

I confirm that I have read this submission and believe that I have an appropriate level of expertise to confirm that it is of an acceptable scientific standard, however I have significant reservations, as outlined above.

# Reviewer report: Reviewer response for version 1

**DOI:** 10.21956/openreseurope.16311.r31477

**Published:** 2023-05-22

**Authors:** Sara Cotterall

**Affiliations:** 1 School of Linguistics and Applied Language Studies, Victoria University of Wellington, Wellington, Wellington, New Zealand

This essay addresses a critical issue that affects large numbers of PhD candidates and Early Stage Researchers (ESRs) worldwide: a marked increase in the levels of anxiety, stress and other mental health issues they experience. Given the high stakes nature of doctoral research, however, this issue is not always adequately acknowledged or addressed by research institutions. Consequently, research into ways of supporting the personal development of doctoral researchers together with their existing research skills training is essential.

 However, personal development training of the type reported in this essay is not the only means of providing support. Other approaches include encouraging ESRs to establish and participate in (online) support groups (Panayidou & Priest, 2021 ^1^; Elliot & Makara, 2021 ^2^; Pretorious *et. al*., 2019 ^3^), enhancing the supervisory relationship (Wollast *et al.,* 2023 ^4^), and using writing networks to build confidence, boost motivation and support identity building (Aitchison & Guerin, 2014 ^5^). It would have been good to locate the approach adopted in this study in the context of other well-researched approaches.

 The account provided of the intervention delivered in this study raises a doubt about the extent to which the training was aligned with the particularities of the doctoral researchers’ contexts. First, the analysis and change model (Bateson, 1972; Dilts, 1996) around which the intervention was organised did not appear to relate specifically to the specialised word of academia. The stress experienced by many doctoral candidates (and junior members of academic research teams) is triggered by factors such as the pressure to publish, funding issues, crises of personal confidence, power dynamics in supervision or research teams, time management problems, intercultural communication concerns, personal financial issues and family roles and responsibilities. Many of these stressors are peculiar to the academic research environment, and to the identity-related aspects of becoming a scholar. Consequently, they warrant particular attention.

 Second, it is difficult to determine the precise **nature** of the intervention because few details are provided. Instead, the bulk of the essay is devoted to reporting the **content** of the programme, detailing the seven very broad Actions (e.g., Discover your purpose in life) recommended in the Personal Development Plan. It would have been helpful to see an example of how one of these Actions had been presented, explored and trialled (?) in the participating ESRs’ respective research contexts.

 This paper would be more valuable for early stage researchers, their supervisors and more senior colleagues and researchers in the field of doctoral education if it were developed along the following lines:

1. Provide examples of the online learning tasks that formed part of the PDP intervention;
2. Include details of the action learning projects that participants were invited to complete;
3. Present sample materials from the wellbeing modules.
4. Incorporate feedback from some/all of the ESRs who completed the programme.

In relation to the final point above, I was left wondering what the 14 Early Stage Researchers thought about the intervention. In other accounts of interventions to support ESRs’ wellbeing, the PhD candidates’ voices are often allocated as much space as the authors’ description of the intervention itself. In the absence of participant evaluations, in this case it is difficult to judge the extent to which the programme met the interpersonal needs of the participants.

Is the argument persuasive and supported by appropriate evidence?

Partly

Is the work clearly and cogently presented?

Yes

Is the topic of the essay discussed accurately in the context of the current literature?

Partly

Does the essay contribute to the cultural, historical, social understanding of the field?

Partly

Reviewer Expertise:

Doctoral education, Higher education, Language teacher education, Language learner autonomy, Language learning strategies

I confirm that I have read this submission and believe that I have an appropriate level of expertise to confirm that it is of an acceptable scientific standard, however I have significant reservations, as outlined above.

## References

[1] : Enhancing postgraduate researcher wellbeing through support groups. *Studies in Graduate and Postdoctoral Education* .2021;12(1) : 10.1108/SGPE-06-2020-0038 42-57 10.1108/SGPE-06-2020-0038

[2] : An online community of international scholars: Enabling spaces for reciprocal academic and psychological support. *Innovations in Education and Teaching International* .2021;58(6) : 10.1080/14703297.2021.1991424 693-703 10.1080/14703297.2021.1991424

[3] : Wellbeing in Doctoral Education: Insights and Guidance from the Student Experience. *Springer Singapore* .2019; 10.1007/978-981-13-9302-0 10.1007/978-981-13-9302-0

[4] : Facing the dropout crisis among PhD candidates: the role of supervisor support in emotional well-being and intended doctoral persistence among men and women. *Studies in Higher Education* .2023; 10.1080/03075079.2023.2172151 1-16 10.1080/03075079.2023.2172151

[5] : Writing Groups for Doctoral Education and Beyond: Innovations in practice and theory. *Routledge* .2014;
